# A Case of Spontaneous Spinal Subdural Hematoma Complicated by Cranial Subarachnoid Hemorrhage and Spinal Adhesive Arachnoiditis

**DOI:** 10.1155/2019/7384701

**Published:** 2019-03-13

**Authors:** Taihei Go, Toshiyuki Tsutsui, Yasuaki Iida, Katsunori Fukutake, Ryoichi Fukano, Kosei Ishigaki, Kazuaki Tsuchiya, Hiroshi Takahashi

**Affiliations:** ^1^Department of Orthopedic Surgery, Sagamihara Chuo Hospital, Kanagawa, Japan; ^2^Department of Orthopedic Surgery, Toho University, Tokyo, Japan

## Abstract

A 76-year-old woman with a spinal subdural hematoma (SDH) was presented with severe back pain without headache. Magnetic resonance imaging (MRI) performed 4 days after onset showed SDH extending from Th2 to L3. She was diagnosed with spontaneous SDH without neurological manifestation, and conservative treatment was selected. Transient disturbance of orientation appeared 7 days after onset. Small subarachnoid hemorrhage (SAH) was detected on head CT, and strict antihypertensive therapy was started. Symptoms changed for the better. Back pain disappeared 4 weeks after onset. On follow-up MRI at 6 months after onset, the SDH had been resolved spontaneously. Although adhesive arachnoiditis was observed at Th4-6, the recurrence of clinical symptoms was not observed at one year and a half after onset. Spinal subdural space is almost avascular; a hematoma in a subdural space is considered to come from a subarachnoid space when it is a lot. A hemorrhage in subarachnoid space was flushed by cerebral spinal fluid; hematoma or arachnoiditis was not formed in general. In our case, hemorrhage was a lot and expansion of SDH was large enough to cause cranial SAH and arachnoiditis. But longitudinally expanded SDH did not show neurological manifestation and resolved spontaneously in our case.

## 1. Introduction

Spinal subdural hematoma (SDH) with no inducer is rarely reported. However, the increased use of magnetic resonance imaging (MRI) has simplified diagnosis of spontaneous SDH with very mild neurologic manifestation, and cases with a favorable outcome achieved by conservative treatment have been reported [1–7]. We encountered a patient with spontaneous SDH complicated by cranial SAH in whom remission was achieved by conservative treatment. Here, we report the case with a literature review.

## 2. Case Presentation

The patient was a 76-year-old woman with a chief complaint of backache. Her medical history included hypertension and lumbar spinal canal stenosis that had not been treated with an oral anticoagulant or antiplatelet agent. She became aware of a sense of discomfort in the dorsal region without cause 4 days before she visited our hospital. Backache aggravated suddenly, and she had vomiting and difficulty with body movement; she visited the Department of Surgery at our hospital and was admitted for examination and treatment. There were no abnormal findings on thoracoabdominal CT or endoscopy from a surgical perspective, and she was referred to our department.

In the initial examination, body temperature was 36.2°C, blood pressure 192/109 mmHg, and pulse 79/min. The consciousness level was Glasgow Coma Scale (GCS) (E4, V4, M6), showing mild disturbance of orientation. She complained severe backache without headache. On neurological examination, no hypesthesia or muscle weakness of the lower limbs was noted. Regarding deep tendon reflexes, both the patellar tendon and Achilles tendon reflexes were (+) on the bilateral sides, showing no increase or reduction, and there was no pathological reflex or bladder and rectal disturbance. There were no other abnormalities, including in hemorrhage and coagulation test findings.

**Figure 1:**
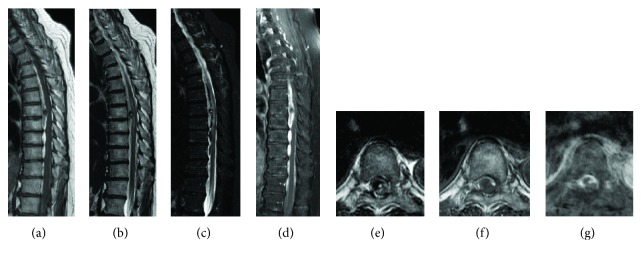
Sagittal (T1-weighted (a), T2-weighted (b), STIR (c), and contrast-enhanced (d)) and axial (T1-weighted (e), T2-weighted (f), and contrast-enhanced (g)) magnetic resonance imaging revealed a large subdural hematoma extending from T2 to L3 and compressing the spinal cord from the ventral side at 7 days after onset.

On plain radiography at admission, there were no abnormal findings in the thoracolumbar vertebrae. On lumbar spinal MRI 4 days after onset, a band-like shadow continuous from the thoracic spinal level with high intensity on T1-weighted imaging and low intensity on T2-weighted imaging, and STIR was detected on the subdural extramedullary ventral side. To examine the lesion at the upper level more closely, thoracic spinal MRI was performed 7 days after onset and a band-like shadow extending from Th2 to L3 was observed on the subdural extramedullary ventral side. A mass present in the shadow at Th7 was compressing and deforming the spinal cord centered on this region, and changes in intramedullary brightness of Th6 over Th8 were noted (Figure 1). There was no tumorous contrast enhancement in the mass region on contrast MRI, and no vascular malformation was observed on contrast-enhanced CT.

**Figure 2:**
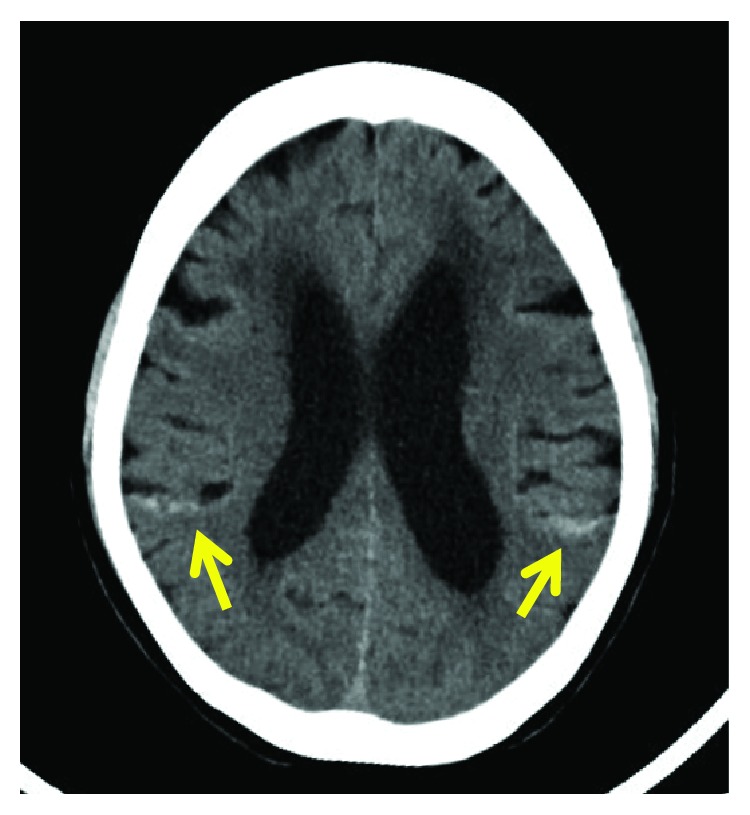
Axial noncontrast brain computed tomography showing a subarachnoid hemorrhage in the bilateral hemispheres at 7 days after onset.

The patient had no previous trauma, abnormality of the coagulation system, or history of lumbar puncture or anticoagulant therapy, and no tumorous lesion or vascular malformation was detected on imaging. Based on these findings, she was diagnosed with spontaneous SDH with no inducer. Since hematoma was extensive and there was no neurologic manifestation, course of observation with conservative treatment was selected. Disturbance of orientation and delusion appeared 7 days after onset, and muscle weakness of MMT 3-4 was observed in the iliopsoas and lower muscles. Cranial SAH was detected in the bilateral parietal lobes and cerebral sulcus in the left occipital lobe on head CT (Figure 2). The hemorrhagic regions differed from the representative hemorrhagic regions of aneurysm rupture and hypertensive hemorrhage, and the hemorrhage volume was small. There was no apparent aggravation, such as expansion of the hematoma and exclusion of the dural canal, on thoracic spinal MRI. Therefore, after consultation with the Department of Neurosurgery, antihypertensive management and intracranial pressure management by intravenous drip infusion were initiated. No aneurysm was detected in screening using head MRA, and the symptoms gradually improved.

**Figure 3:**
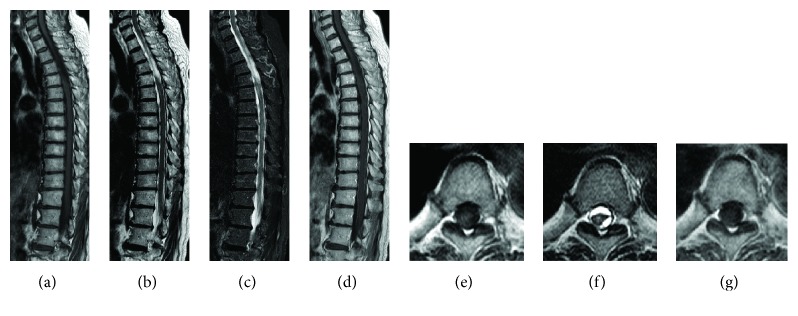
Sagittal (T1-weighted (a), T2-weighted (b), STIR (c), and contrast-enhanced (d)) and axial (T1-weighted (e), T2-weighted (f), and contrast-enhanced (g)) magnetic resonance imaging showed resolution of the subdural hematoma and deformity of the spinal cord indicating adhesive arachnoiditis at 6 months after onset.

Muscular strength of the iliopsoas and lower muscles recovered to MMT 4-5 at 3 weeks after onset, and backache and disturbance of orientation resolved at 4 weeks, after which ambulation was started. Muscular strength of the iliopsoas and lower muscles recovered to a normal level of MMT 5 at 6 weeks after onset, and the patient transferred to a rehabilitation hospital at 7 weeks. She was able to walk with canes after 6 months and was discharged home. On MRI at 6 months after onset, the hematoma had been absorbed, the mass had shrunk, and changes in intramedullary brightness had been reduced. However, dilation of the subarachnoid space at Th4-6 and displacement and deformity of the spinal cord were observed (Figure 3). These findings were considered to be due to adhesive arachnoiditis, but the course remained favorable thereafter. No recurrence of backache or neurologic manifestation was noted at final follow-up at one year after onset.

## 3. Discussion

Cases of SDH often occur because of trauma, abnormal blood coagulation, and iatrogenic causes such as anticoagulant therapy and lumbar puncture and are occasionally caused by vascular malformation and tumorous lesions. However, spontaneous SDH is rare, with a rate of 14% in a review of 106 cases of nontraumatic SDH by Domenicucci et al. [8]. To our knowledge, a total of 45 spontaneous cases have been reported [9]. The functional outcome has been poor in many cases complicated by SAH, but diagnosis of SDH with very mild neurologic manifestation has become easier using MRI, and mild cases with favorable outcomes after conservative treatment have recently been increasingly reported [1–7].

SDH initially occurs with sudden low back pain in many cases, with manifestation of motor paralysis, paresthesia, and autonomic disorder. In imaging, the lesion frequently develops at the thoracic over the lumbar spinal level, and hematoma expansion in the craniocaudal direction can vary from one to 19 vertebrae [9]. Hematoma develops on the ventral and dorsal sides of the spinal cord, but the frequency on the ventral side is slightly higher [9]. Our patient mostly showed these typical imaging findings. Conservative treatment is selected for cases with very mild neurologic manifestation and a tendency for improvement, whereas surgical treatment is used for cases with serious symptoms and acute progression [9–11].

The source of bleeding of spinal subdural hemorrhage is considered to be blood vessels in the subarachnoid space because blood vessels in the subdural space are minute. The mechanism of hematoma formation is thought to be due to an initial intrathoracic or intraperitoneal pressure increase in response to very mild trauma. This ruptures a vein at the spinal root without a valve structure distributed in the subarachnoid space, and hemorrhage breaks the arachnoid and flows into the subdural space, forming a hematoma [12]. Generally, hemorrhage in the subdural space does not form subarachnoid hematoma or cause adhesive arachnoiditis because it is diluted with cerebrospinal fluid (CSF), and the fibrinolysis system is activated [13, 14].

In our patient, a small volume of SAH was observed on the bilateral sides on head CT at one week after onset. To date, there have been only two cases of simultaneous spinal SDH and cranial SAH in the previous literatures. There are three theories regarding simultaneous spinal SDH and cranial SAH. The first is coincidentally development of spinal SDH and cranial SAH under different mechanisms, which is quite rare. The second is when there is a cranial SAH first and then it spreads to the spine. The hemorrhage breaks down subarachnoid membrane in the spine and turns into SDH. The last one is a reverse form of the second mechanism, from spinal SAH and SDH to cranial SAH [15, 16]. So far, it is widely believed that there are minute vessels in spinal subdural space; large amounts of spinal SAH can develop spinal SDH and cranial SAH. In our patient, the initial symptom was backache without headache at onset, and MRI showed spinal SDH. Head symptoms developed one week later, and CT showed small amount of cranial SAH then. Additionally, arachnoiditis was found at Th4-6 six months later, which was found to have a large amount of spinal SDH. This is a clear clue that spinal SDH began as spinal SAH initially, which is believed to have spread out through arachnoid membrane. Because the subarachnoid space in the spinal cord is smaller than in the head, it is usually diagnosed as spinal SDH initially. It is difficult to distinguish the SAH in the early stages of a large amount of SDH.

In our patient, no hemorrhage was detected on head CT in the basilar cistern or sylvian fissure, the inflow route from the spinal cord to the head. In a case report of spinal subarachnoid hematoma, there was similarly no hemorrhage detected in the basilar cistern or sylvian fissure, with a small volume of SAH observed only in the median parietal region [17]. It was suggested that the hemorrhage in the subarachnoid space may have been diluted with CSF and that this dilution may have occurred to a degree that made the hemorrhage undetectable by head CT when it reached the intracranial region; then, the concentration increased around the arachnoid granulation, in which CSF is absorbed, resulting in detection on CT [17]. In our patient, the concentration may also have increased in the same region through this mechanism and resulted in meningeal irritation, as well as detection of SAH on head CT.

The neurologic manifestation in our patient was very mild. This may have been because hemorrhage flowing into the subdural space expanded mainly in the craniocaudal direction, which reduced local spinal cord compression. However, such long expansion in this direction may have caused compression by hematoma and CSF reperfusion injury, inducing adhesive arachnoiditis. There is only one other reported case with concomitant adhesive arachnoiditis that developed after spontaneous absorption of SDH [18]. In this case, hematoma expanded to the Th10 over S1 level and neurologic manifestation was very mild without motor paralysis, as in our patient; therefore, conservative treatment was selected. However, a spinal arachnoid cyst complicating adhesive arachnoiditis developed 3 months after onset and neurologic manifestation aggravated, for which syringo-peritoneal (S-P) shunt was performed [18]. In our patient, the course was favorable at one year after onset, but continued attention to possible aggravation of neurologic manifestation is required.

Complications of SAH in the head include late-onset cerebral vasospasm. This develops following irreversible stenosis of a cerebral major artery 4-14 days after onset of SAH. Definite diagnosis is made using cerebral angiography. The risk of cerebral vasospasm is proportional to the volume of cisternal hemorrhage, with a low risk in cases with a small hemorrhage volume, such as that in our patient. However, Shakur and Farhat detected cerebral vasospasm-induced cerebral infarction 5 days after onset in cases of spontaneous spinal subarachnoid hematoma with a small volume of SAH in the median parietal region only [19]. Production of a large quantity of oxyhemoglobin, the causative substance of cerebral vasospasm, induced by hemolysis in the spinal cord medullary cavity was suggested to be the cause [19].

In our patient, head symptoms were noted one week after admission, but it is unclear whether these symptoms were due to SAH-induced cerebral vasospasm because cerebral angiography was not performed. However, in cases with extensive SDH, SAH symptoms induced by inflow of hemorrhage into the head and cerebral vasospasm-induced symptoms developing through a mechanism similar to that reported by Shakur and Farhat may develop. In such cases, a treatment approach that includes consultation with the Department of Neurosurgery is important.

## 4. Conclusion

We encountered a patient with spontaneous SDH with backache and vomiting at onset that was resolved by conservative treatment. For hemorrhage in the spinal subarachnoid space, attention should be paid to possible inflow of hemorrhage into the head. If head symptoms develop, rapid examination by head CT and consultation with the Department of Neurosurgery are important. In our patient, adhesive arachnoiditis associated with SAH developed. Careful course observation is continuing in this patient.
